# Inhibition of Sphingosine-1-Phosphate Receptor 2 by JTE013 Promoted Osteogenesis by Increasing Vesicle Trafficking, Wnt/Ca^2+^, and BMP/Smad Signaling

**DOI:** 10.3390/ijms222112060

**Published:** 2021-11-08

**Authors:** Simon Lin, Subramanya Pandruvada, Hong Yu

**Affiliations:** Department of Oral Health Sciences, College of Dental Medicine, Medical University of South Carolina, Charleston, SC 29425, USA; linsi@musc.edu (S.L.); pandruv@musc.edu (S.P.)

**Keywords:** S1PR2, JTE013, osteogenesis, matrix vesicle, vesicle trafficking, Wnt, calcium, BMP

## Abstract

Sphingosine-1-phosphate receptor 2 (S1PR2) is a G protein-coupled receptor that regulates various immune responses. Herein, we determine the effects of a S1PR2 antagonist (JTE013) or a S1PR2 shRNA on osteogenesis by culturing murine bone marrow stromal cells (BMSCs) in osteogenic media with JTE013, dimethylsulfoxide (DMSO), a S1PR2 shRNA, or a control shRNA. Treatment with JTE013 or the S1PR2 shRNA increased alkaline phosphatase and alizarin red s staining, and enhanced alkaline phosphatase, RUNX2, osteocalcin, and osterix mRNA levels in BMSCs compared with the controls. Protein analysis revealed that a high dose of JTE013 (4 or 8 μM) increased vesicle trafficking-associated proteins (F-actin, clathrin, Early Endosome Antigen 1 (EEA1), and syntaxin 6) and Wnt/Ca2+ signaling. On the other hand, a low dose of JTE013 (1 to 2 μM) increased BMP/Smad signaling. In contrast, the S1PR2 shRNA reduced vesicle trafficking-associated proteins and attenuated Wnts and BMP/Smad signaling, but enhanced p-CaMKII compared with the control, suggesting that the S1PR2 shRNA influenced osteogenesis via different signaling pathways. Moreover, inhibiting protein trafficking by brefeldin A in BMSCs suppressed Wnts and BMPRs expressions. These data supported that enhanced osteogenesis in JTE013-treated BMSCs is associated with increased vesicle trafficking, which promotes the synthesis and transport of osteogenic protein and matrix vesicles and enhances matrix mineralization.

## 1. Introduction

Sphingosine-1-phosphate (S1P) is a bioactive sphingolipid, which can be generated by activation of sphingosine kinases with various stimuli, including growth factors, hormones, bacterial stimuli, and cytokines [1,2]. Because S1P can be degraded by S1P lyase or dephosphorylated by S1P phosphatases in the tissues, the constitutive S1P level in tissues is very low (10–30 nM) [3]. In contrast, the S1P level in the blood is very high (150–1000 nM) because platelets lack S1P lyase and erythrocytes lack both S1P lyase and S1P phosphatases [3,4]. This sharp gradient between the S1P level in the blood and the S1P level in the tissues controls the migration of immune cells from blood to peripheral tissues, subsequently influencing the inflammatory response in the tissues [3,4].

S1P plays multifaceted roles in bone homeostasis. First, S1P controls monocytes (osteoclast precursors) migration from the blood to bone tissues, subsequently affecting osteoclastogenesis [5,6]. Second, S1P promotes osteoblast chemotaxis and survival, subsequently increasing RANKL production and affecting osteoclastogenesis [7]. Higher circulating S1P levels were associated with lower bone mineral density and higher bone resorption markers in postmenopausal women [8]. Third, under physiological condition, S1P can also promote osteoblast proliferation. Deficiency in S1P lyase in mice or pharmacological inhibition of S1P lyase led to an increased in the S1P level, and markedly enhanced bone mass and strength in animals [9].

S1P binds to five G protein-coupled S1P receptors (S1PR1–5). S1PR1 is coupled to G_i_; S1PR2 and S1PR3 are coupled to G_i_, G_q_, and G_12/13_ proteins; whereas S1PR4 and S1PR5 are coupled chiefly to G_i_ and G_12/13_ in response to S1P [1]. S1PR2 regulates various cellular signaling pathways, including adenylate cyclase, phospholipase C (PLC), phosphoinositide-3 kinase (PI3K), nuclear kappa-B (NF-κB), extracellular signal-regulated kinase (ERK), c-Jun N-terminal kinase (JNK), p38 mitogen-activated kinase (MAPK), and small G proteins Rac and Rho [1,10,11,12]. Previously, there were conflicting results regarding how S1PR2 regulates osteogenesis. Ishii et al. [13] demonstrated that a strain of S1PR2-deficient mice increased bone volume, trabecular thickness, and trabecular density compared with wild-type mice. However, Higashi et al. [14] showed that a high concentration of S1P (2 μM) enhanced RUNX2 expression by promoting S1PR2/RhoA/ROCK/Smad1/5/8 signaling. Inhibition of S1PR2 by a S1PR2 antagonist, JTE013, reduced RUNX2 induced by S1P [14]. They also showed that a S1PR2 agonist, CYM5520, enhanced trabecular bone volume in animals [14]. Another study [15] demonstrated that treatment with a low dose of JTE013 (1 μM) increased the proliferation of murine BMSCs by enhancing p-ERK expression. However, treatment with JTE013 (1 μM) reduced osteopontin expression in murine BMSCs [15].

Additionally, previous studies demonstrated that S1PR2 not only controls S1P signaling, but also regulates many other signaling pathways induced by other factors [16,17,18,19]. Our studies showed that treatment with a S1PR2 shRNA, or the specific S1PR2 antagonist, JTE013, suppressed PI3K, MAPKs, and NF-κB protein kinases induced by an oral bacterial pathogen *Aggregatibacter actinomycetemcomitans*, subsequently reduced inflammatory cytokines (IL-1β, IL-6, and TNF-α) levels in murine bone marrow-derived monocytes and macrophages (BMMs) compared with controls [20,21]. Additionally, treatment with the S1PR2 shRNA or JTE013 decreased podosome components (cell adhesion structural units, including PI3K, Src, Pyk2, integrin β3, F-actin, and paxillin levels) induced by RANKL, resulting in the inhibition of osteoclastogenesis induced by RANKL compared with the controls [21]. In contrast, S1P (<1 μM) could not induce a significant inflammatory cytokine production in BMMs [22], and treatment with S1P (1 to 1000 nM) in a single culture of murine BMMs did not affect RANKL-induced osteoclast differentiation [7]. In animal studies, treatment with JTE013 in mice alleviated periodontal inflammatory bone loss induced by ligature placement [23] and attenuated osteoporosis induced by RANKL [13]. Moreover, in a streptozotocin-induced type I diabetes animal model, treatment with JTE013 attenuated streptozotocin-induced apoptosis of pancreatic beta cells and decreased the incidence of diabetes [17]. Additionally, in a high-fat die-induced type II diabetes animal model, treatment with JTE013 improved glucose tolerance and increased insulin sensitivity in mice [18]. These studies demonstrated that S1PR2 modulates many cell signaling pathways that are induced by many factors.

Bone is generated by osteoblasts, which are derived from mesenchymal stem cells. Mesenchymal stem cells undergo cell proliferation, synthesize bone matrix proteins, and deposit minerals on bone matrix, forming bone tissues. Osteoblasts synthesize very dense and cross-linked collagen, osteogenic enzymes, including alkaline phosphatase (ALP), and proteins, including Wnts, bone morphogenetic proteins (BMPs), osteocalcin (OCN), and osterix (OSX). Additionally, osteoblasts produce calcium and phosphate-based minerals, which are deposited into the organic matrix, forming mineralized bone tissues.

Osteogenesis is regulated by various signaling pathways, including the Wnt and BMPs signaling pathways [24,25,26,27,28]. The Wnt signaling pathways can be classified as Wnt canonical and Wnt non-canonical pathways. The Wnt canonical pathway is mediated by β-catenin. In the absence of Wnt, cytoplasmic β-catenin is constantly degraded by the Axin complex, which is composed of the scaffolding protein Axin, the tumor suppressor adenomatous polyposis coli gene product (APC), casein kinase 1 (CK1), and glycogen synthase kinase 3 (GSK3). CK1 and GSK3 sequentially phosphorylate the amino terminal region of β-catenin, resulting in β-catenin recognition by β-Trcp, an E3 ubiquitin ligase subunit, and subsequent β-catenin ubiquitination and proteasomal degradation [29]. In the presence of Wnt, Wnt ligands bind to a complex of the seven-pass transmembrane Frizzled (Fz) receptor and low-density lipoprotein receptor–related protein (LPR) 5/6. The formation of the Wnt-Fz-LPR5/6 complex, together with the recruitment of scaffolding protein Disheveled (Dvl), results in LPR5/6 phosphorylation and the recruitment of the Axin complex to the receptors [29]. These events inhibit the degradation of β-catenin in the cytoplasm, resulting in accumulation of β-catenin in the cytoplasm and followed by β-catenin nuclear translocation [25,30]. The Wnt non-canonical pathways are classified as the Wnt/planar cell polarity (PCP) pathway and the Wnt/Ca^2+^ pathway. In the Wnt/PCP pathway, Wnt ligands bind to Fz receptors that activate small GTPase Rac1, RhoA, and downstream JNK, which control the rearrangements in the cytoskeleton and gene expression [25,30]. In the Wnt/Ca^2+^ pathway, Wnt ligands bind to Fz receptors, which activate heterotrimeric G proteins and PLC [31,32]. Activation of PLC triggers the generation of inositol 1,4,5-triphosphate (IP3) and 1,2-diacylglycerol (DAG) [31,32]. IP3 diffuses through the cytosol and interacts with the calcium channels on the membrane of endoplasmic reticulum (ER), resulting in the release of Ca^2+^ [31,32]. Ca^2+^ subsequently activates the calcium/calmodulin-activated protein kinase (CaMK)II [31,32]. Ca^2+^ and DAG can further activate protein kinase C (PKC) [31,32].

The BMPs signaling involves BMPs dimers that bind to BMP type II receptors, which heterodimerize with type I receptors and subsequently activate the receptor-regulated (R)-Smads (Smad 1, 5, and 8). Phosphorylation of Smad 1, 5, and 8 heterodimers form a complex with the only common mediator, (Co)-Smad (Smad4). Following nuclear translocation of the R-Smad/Co-Smad complex, the complex activates multiple transcriptional factors, including RUNX2 [27,28,33]. BMPs can also activate MAPKs, which in turn promote RUNX2 expression [33,34].

Additionally, osteoblasts possess matrix vesicles (MVs), which are extracellular membrane-bounded microparticles, composed of cytoskeletal and surface proteins (including actin and integrins), as well as proteins and enzymes involved in synthesis and transport of Ca^2+^ and PO4^3−^, including tissue non-specific alkaline phosphatase (TNAP), phosphoethanolamine/phosphocholine phosphatase (PHOSPHO-1), annexins, and phospholipases [35,36,37,38,39]. MVs also possess phospholipids, clathrin heavy chain, syntaxin, and G proteins [38,39]. The major function of MVs is the initiation of mineralization. Previous studies revealed that apatite initially is formed within MVs. Then, apatite platelets propagate and are inserted into collagen fibrils, leading to matrix mineralization [35,36,37,38,39]. The biosynthesis of MVs is involved with the endosomal pathway via exocytosis [38], and the actin network is extensively involved during MVs formation [40,41].

Vesicle trafficking is involved in both endocytosis and exocytosis. Endocytosis is coupled with exocytosis because endocytosis maintains exocytosis via recycling vesicles to prevent vesicle exhaustion [42]. Vesicle trafficking is regulated by G proteins, including Rac [43,44]. Rac activates the Wiskott–Aldrich syndrome protein family verprolin-homologous protein (WAVE) [43,44]. Activation of WAVE leads to the formation of the actin-related protein 2/3 (Arp2/3) complex, subsequently resulting in polymerization of actin, which polymerizes globular (G) actin monomers to form filament (F)-actin [43,44]. Polymerization of actin subsequently promotes vesicle trafficking [43,44]. Previous studies have demonstrated that S1PR2 inhibits Rac activity [11,12]; also, S1PR2 deficiency in murine bone marrow-derived macrophages enhanced Rac1-GTP and F-actin stimulated by *Escherichia coli* (*E. coli*), leading to increased bacterial phagocytosis [19]. Treatment with the S1PR2 antagonist, JTE013, also promoted bacterial clearance and improved survival in mice challenged with *E. coli* [19]. In this study, we treated murine bone marrow-derived stromal cells (BMSCs) with control vehicle DMSO, JTE013 (0.5 to 8 μM), a control shRNA, or a S1PR2 shRNA to determine the effects of JTE013 or the S1PR2 shRNA on osteogenesis.

## 2. Results

### 2.1. JTE013 Affects Many S1PRs Expressions in Murine BMSCs

**Figure 1:**
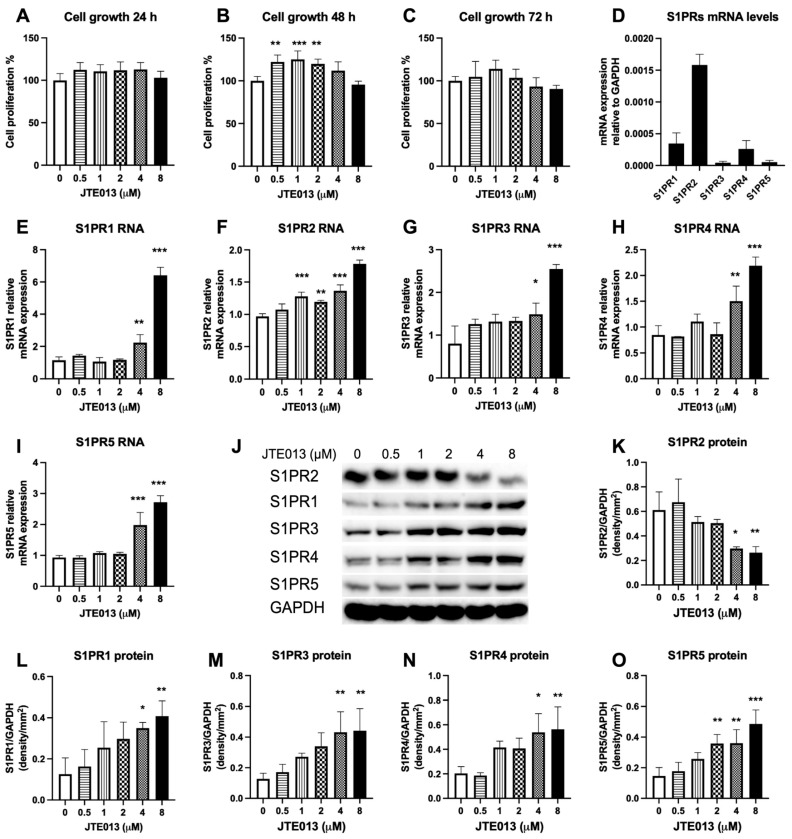
Effects of JTE013 on cell growth and S1PRs expressions. Murine BMSCs were cultured in osteogenic media for 1 to 3 days with the presence of either DMSO or JTE013 (0.5 to 8 μM). Cell growth at (**A**) 24 h, (**B**) 48 h, and (**C**) 72 h was quantified by a CellTiter 96^®^ AQueous One Solution Cell Proliferation Assay. (**D**) Relative S1PRs’ mRNA levels in murine BMSCs cultured in DMEM media with 10% FBS and 100 U/mL penicillin and streptomycin. (**E**–**I**) Murine BMSCs were treated with either DMSO or JTE013 (0.5 to 8 μM) and cultured in osteogenic media for 7 days. (**E**) S1PR1, (**F**) S1PR2, (**G**) S1PR3, (**H**) S1PR4, and (**I**) S1PR5 mRNA levels were quantified by RT-qPCR and normalized by GAPDH expression. (n = 3, * *p* < 0.05, ** *p* < 0.01, *** *p* < 0.001). (**J**–**O**) Murine BMSCs were treated with either DMSO or JTE013 (0.5 to 8 μM) and cultured in osteogenic media for 9 days. (**J**) S1PR2, S1PR1, S1PR3, S1PR4, S1PR5, and GAPDH protein levels were measured by Western blot. Protein densitometry of (**K**) S1PR2, (**L**) S1PR1, (**M**) S1PR3, (**N**) S1PR4, and (**O**) S1PR5 was normalized by GAPDH protein expression in BMSCs (n = 3, * *p* < 0.05, ** *p* < 0.01, *** *p* < 0.001).

First, we evaluated the effect of JTE013 on cell growth in BMSCs. In murine BMSCs treated with various doses of JTE013 for 24 h, lower doses of JTE013 (0.5 to 4 μM) increased cell growth by about 10.6% to 12.8% compared with DMSO treatment group. However, there were no significant differences among treatment groups (Figure 1A). We observed a significant increase of cell growth in murine BMSCs treated with lower doses of JTE013 for 48 h (Figure 1B). JTE013 (0.5, 1, and 2 μM) increased cell growth by about 22.0%, 25.1%, and 19.8%, respectively, compared with DMSO treatment. However, 72 h after treatment, there were no significant differences of cell growth among treatment groups (Figure 1C). The highest dose of JTE013 (8 μM) slightly reduced cell growth by about 9.5% at 72 h compared with DMSO treatment (Figure 1C).

Murine BMSCs express all five S1PRs’ mRNA (Figure 1D), with S1PR2 expressed at the highest level, which was about 4.6-fold of S1PR1, 35.7-fold of S1PR3, 6.1-fold of S1PR4, and 30.5-fold of S1PR5. Treatment with a high dose of JTE013 (4 or 8 μM) significantly increased all S1PRs’ mRNA levels (Figure 1E–I). Treatment with a high dose of JTE013 (4 or 8 μM) significantly reduced S1PR2 protein expression in BMSCs (Figure 1J,K). In contrast, a low dose of JTE013 (0.5 to 2 μM) had no significant effect on S1PR2 protein expression. Treatment with a high dose of JTE013 (4 or 8 μM) also significantly increased S1PR1, S1PR3, S1PR4, and S1PR5 protein levels (Figure 1L–O). These results demonstrated that a high dose of JTE013 (4 or 8 μM) reduced S1PR2 protein expression, but increased S1PR1, S1PR3, S1PR4, and S1PR5 mRNA and protein expressions in BMSCs.

### 2.2. Treatment with JTE013 or a S1PR2 ShRNA Increased ALP Staining in Murine BMSCs

**Figure 2:**
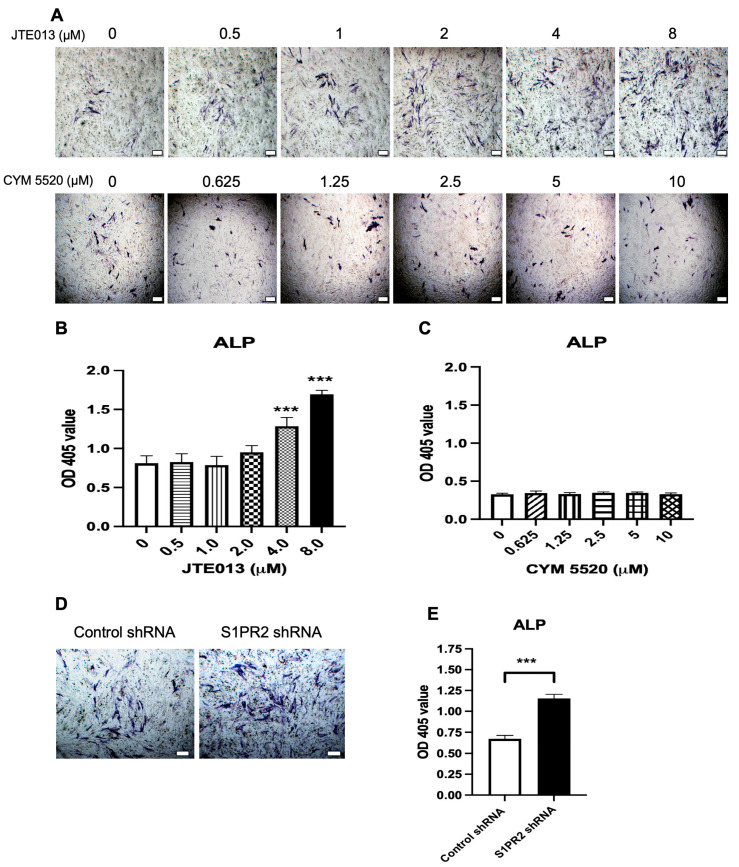
Effects of a S1PR2 antagonist (JTE013), a S1PR2 agonist (CYM 5520), a control shRNA, or a S1PR2 shRNA on alkaline phosphatase (ALP) staining in murine BMSCs. Murine BMSCs were cultured in osteogenic media with either vehicle (DMSO), JTE013 (0.5 to 8 μM), CYM5520 (0.625 to 10 μM), a control shRNA, or a S1PR2 shRNA for 7 days. (**A**) Representative images of ALP staining in murine BMSCs treated with DMSO, JTE013, or CYM5520. (**B**) Quantification of ALP staining in murine BMSCs treated with DMSO or JTE013 (n = 3, *** *p* < 0.001). (**C**) Quantification of ALP staining in murine BMSCs treated with DMSO or CYM 5520. (**D**) Representative images of ALP staining in murine BMSCs treated with either a control shRNA or a S1PR2 shRNA. (**E**) Quantification of ALP staining in murine BMSCs treated with a control shRNA or a S1PR2 shRNA. All images were taken under 40× magnification. Scale bars represent 200 μm. For quantification of ALP staining, the OD405 value was calibrated by protein concentration in cell protein lysates (n = 3, *** *p* < 0.001).

Next, we determined whether treatment with JTE013 or a S1PR2 shRNA could affect the expression of ALP, an important enzyme in bone mineralization, which hydrolyzes inorganic pyrophosphate (PPi, an inhibitor of mineralization) to inorganic phosphate (Pi) and promotes mineralization [45]. Because a previous study [14] showed that a S1PR2 agonist, CYM5520, enhanced trabecular bone volume in mice, we also analyzed the effect of CYM5520 in ALP expression in BMSCs. As shown in Figure 2A,B, JTE013 dose dependently increased ALP staining in BMSCs. JTE013 (4 and 8 μM) enhanced ALP activity by 1.6-fold and 2.1-fold, respectively, compared with control DMSO treatment. In contrast, treatment with CYM5520 had no significant effect on ALP expression in BMSCs (Figure 2A,C). Treatment with the S1PR2 shRNA increased ALP staining by 1.7-fold compared with BMSCs treated with the control shRNA (Figure 2D,E).

### 2.3. Treatment with JTE013 or a S1PR2 ShRNA Increased Alizarin Red S (ARS) Staining in Murine BMSCs

**Figure 3:**
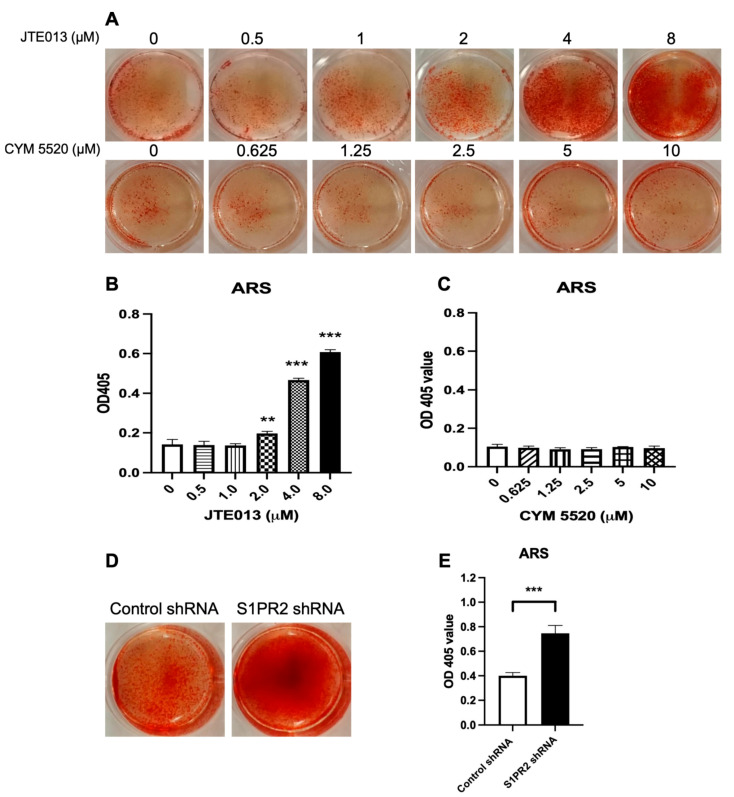
Effects of a S1PR2 antagonist (JTE013), a S1PR2 agonist (CYM 5520), a control shRNA, or a S1PR2 shRNA on alizarin red s (ARS) staining in murine BMSCs. Murine BMSCs were cultured in osteogenic media with either vehicle (DMSO), JTE013 (0.5 to 8 μM), or CYM5520 (0.625 to 10 μM) for 21 days, or cultured with a control shRNA or a S1PR2 shRNA for 14 days. (**A**) Representative images of ARS staining in murine BMSCs treated with DMSO, JTE013, or CYM5520. (**B**) Quantification of ARS staining in murine BMSCs treated with DMSO or JTE013 (n = 3, ** *p* < 0.01, *** *p* < 0.001). (**C**) Quantification of ARS staining in murine BMSCs treated with DMSO or CYM 5520. (**D**) Representative images of ARS staining in murine BMSCs treated with a control shRNA or a S1PR2 shRNA. (**E**) Quantification of ARS staining in murine BMSCs treated with a control shRNA or a S1PR2 shRNA (n = 3, *** *p* < 0.001). For quantification of ARS staining, the OD405 value was calibrated by protein concentration in cell protein lysates.

Next, we determined whether treatment with JTE013, CYM5520, or the S1PR2 shRNA could influence the mineralization of osteoblasts. As shown in Figure 3A, JTE013 dose dependently increased ARS staining in BMSCs. Quantification of ARS staining revealed that JTE013 (2, 4, and 8 μM) enhanced mineralization by 1.4-fold, 3.3-fold, and 4.3-fold, respectively, compared with DMSO treatment. In contrast, treatment with CYM5520 had no effect on mineralization in BMSCs (Figure 3A,C). Treatment with the S1PR2 shRNA also increased ARS staining by 1.9-fold compared with the control shRNA treatment (Figure 3D,E).

### 2.4. Treatment with JTE013 or a S1PR2 ShRNA Increased ALPL, RUNX2, OCN, and OSX mRNA Levels in BMSCs

**Figure 4:**
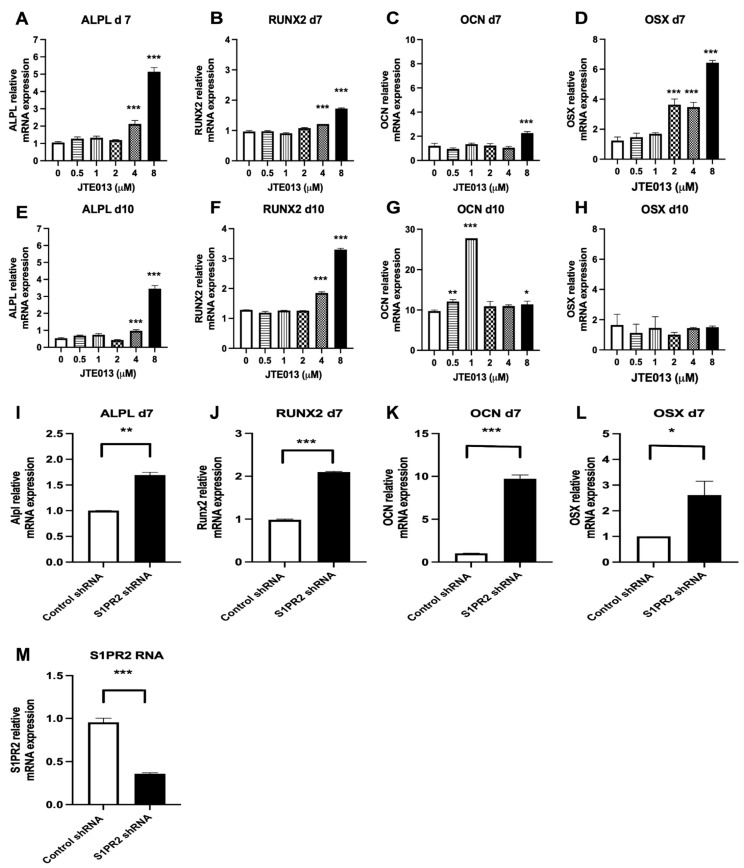
Treatment with the S1PR2 antagonist (JTE013) or a S1PR2 shRNA increased osteogenic gene (ALPL, RUNX2, OCN, and OSX) expressions in murine BMSCs. Murine BMSCs were cultured in osteogenic media with either vehicle (DMSO) or JTE013 (0.5 to 8 μM) for 7 or 10 days, or were cultured in osteogenic media with a control shRNA or a S1PR2 shRNA for 7 days. (**A**,**E**) ALPL, (**B**,**F**) RUNX2, (**C**,**G**) OCN, and (**D**,**H**) OSX mRNA levels in murine BMSCs treated with either DMSO or various doses of JTE013 on day 7 or day 10 were quantified by RT-qPCR and normalized by GAPDH expression. (**I**) ALPL, (**J**) RUNX2, (**K**) OCN, (**L**) OSX, and (**M**) S1PR2 mRNA levels in murine BMSCs treated with either a control shRNA or a S1PR2 shRNA on day 7 were quantified by RT-qPCR and normalized by GAPDH expression. (n = 3, * *p* < 0.05, ** *p* < 0.01, *** *p* < 0.001).

To further determine how JTE013 and the S1PR2 shRNA affect osteogenesis, we analyzed osteogenic genes, including alkaline phosphatase (ALPL), runt-related transcription factor 2 (RUNX2), osteocalcin (OCN), and osterix (OSX) in BMSCs by RT-qPCR. JTE013 (4 μM and 8 μM) significantly increased ALPL mRNA level by 2.0-fold and 4.9-fold on day 7 (Figure 4A) and enhanced ALPL mRNA level by 1.8-fold and 6.4-fold on day 10 (Figure 4E), respectively, compared with control DMSO treatment. JTE013 (4 μM and 8 μM) significantly increased RUNX2 mRNA level by 1.3-fold and 1.8-fold on day 7 (Figure 4B) and enhanced RUNX2 mRNA level by 1.4-fold and 2.6-fold on day 10 (Figure 4F), respectively, compared with control DMSO treatment. OCN, a late osteogenic gene, increased 8.0-fold on day 10 in DMSO-treated BMSCs compared with the OCN level on day 7 in DMSO-treated cells (Figure 4C,G). JTE013 (8 μM) significantly increased the OCN mRNA level by 1.9-fold on day 7 (Figure 4C) and 1.2-fold on day 10 (Figure 4G), respectively, compared with DMSO treatment. We also noticed significant increases in OCN expression on day 10 when treated with lower doses of JTE013 (Figure 4G). JTE013 (0.5 μM and 1 μM) increased OCN on day 10 by 1.2-fold and 2.8-fold, respectively, compared with DMSO treatment. JTE013 (2, 4, and 8 μM) significantly increased OSX mRNA level by 2.9-fold, 2.8-fold, and 5.2-fold on day 7 (Figure 4D), respectively, compared with DMSO treatment. There was no significant difference in OSX mRNA levels among treatment groups on day 10 (Figure 4H). These results demonstrated that higher doses of JTE013 increased mainly early osteogenic marker gene expression, while a low dose of JTE013 enhanced the late osteogenic marker (OCN) expression. Additionally, treatment with the S1PR2 shRNA increased ALPL, RUNX2, OCN, and OSX mRNA levels by 1.7-fold, 2.1-fold, 9.7-fold, and 2.6-fold, respectively, compared with BMSCs treated with the control shRNA (Figure 4I–L). The S1PR2 shRNA reduced S1PR2 mRNA level about 64% compared with control shRNA treatment (Figure 4M).

### 2.5. JTE013, but Not the S1PR2 ShRNA, Increased Vesicle Trafficking-Associated Protein (F-actin, EEA1, Clathrin, and Syntaxin 6) Expressions in Murine BMSCs

**Figure 5:**
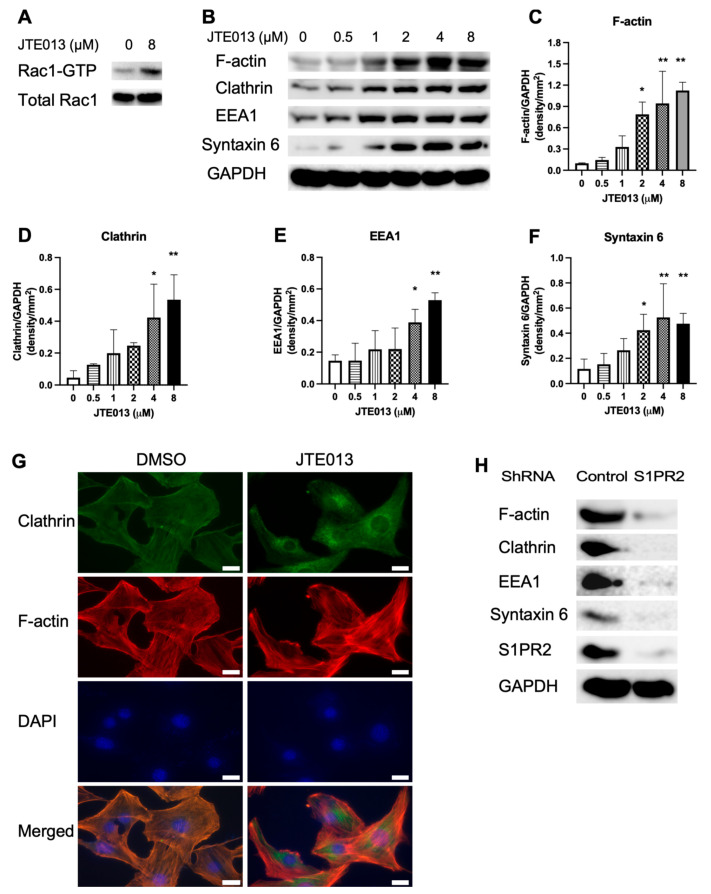
JTE013, but not a S1PR2 shRNA, increased vesicle trafficking-associated protein levels in murine BMSCs cultured in osteogenic media. Murine BMSCs were cultured in osteogenic media with either vehicle (DMSO) or JTE013 for 9 days. Proteins were harvested 20 min (for Rac1) or 30 min (for other proteins) after changing osteogenic media with or without JTE013. (**A**) Rac1-GTP and total Rac1 in BMSCs. (**B**) F-actin, clathrin, EEA1, syntaxin 6, and GAPDH protein levels in murine BMSCs treated with DMSO or various doses of JTE013. Protein densitometry of (**C**) F-actin, (**D**) clathrin, (**E**) EEA1, and (**F**) syntaxin 6 in murine BMSCs treated with DMSO or JTE013 was normalized by GAPDH protein expression in BMSCs (n = 3, * *p* < 0.05, ** *p* < 0.01). (**G**) Representative images of immunofluorescence staining of clathrin, F-actin, and DAPI in murine BMSCs 30 min after changing with osteogenic media. The images were taken under 400× magnification. The scale bars represent 20 μm. (**H**) F-actin, clathrin, EEA1, syntaxin 6, S1PR2, and GAPDH protein levels in murine BMSCs treated with a control shRNA or a S1PR2 shRNA and cultured in osteogenic media for 9 days. Proteins were harvested 30 min after changing with osteogenic media with puromycin (1 μg/mL).

JTE013 was previously shown to increased bacterial endocytosis and phagocytosis [19]. We hypothesized that treatment with JTE013 could enhance Rac1-GTP, which subsequently could promote actin polymerization and vesicle trafficking. As shown in Figure 5A, treatment with JTE013 (8 μM) enhanced Rac1-GTP compared with DMSO treatment in BMSCs. Treatment with JTE013 (2 to 8 μM) significantly enhanced F-actin (Figure 5B,C) and syntaxin 6 (Figure 5B,F) in BMSCs cultured with osteogenic media compared with DMSO treatment (* *p* < 0.05, ** *p* < 0.01). Treatment with JTE013 (4 to 8 μM) also significantly increased clathrin heavy chain (Figure 5B,D) and EEA1 expressions (Figure 5B,E) compared with DMSO treatment (* *p* < 0.05, ** *p* < 0.01). Immunofluoresence staining of clathrin shows that JTE013 (8 μM) induced the expression of clathrin-coated vesicles in the cytoplasm 30 min after changing with osteogenic media compared with BMSCs treated with DMSO and cultured in osteogenic media (Figure 5G). These data suggest that treatment with JTE013 increased vesicle trafficking in murine BMSCs cultured in osteogenic media. However, we observed a reduction of expressions of F-actin, clathrin, EEA1, and syntaxin 6 in murine BMSCs treated with the S1PR2 shRNA compared with murine BMSCs treated with the control shRNA (Figure 5H), which suggests that the S1PR2 shRNA inhibited vesicle trafficking in murine BMSCs cultured in osteogenic media compared with the control shRNA treatment. The S1PR2 shRNA reduced S1PR2 protein expression in murine BMSCs (Figure 5H).

### 2.6. JTE013, but Not the S1PR2 shRNA, Increased RUNX2 and Wnt/Ca^2+^ Signaling in Murine BMSCs

**Figure 6:**
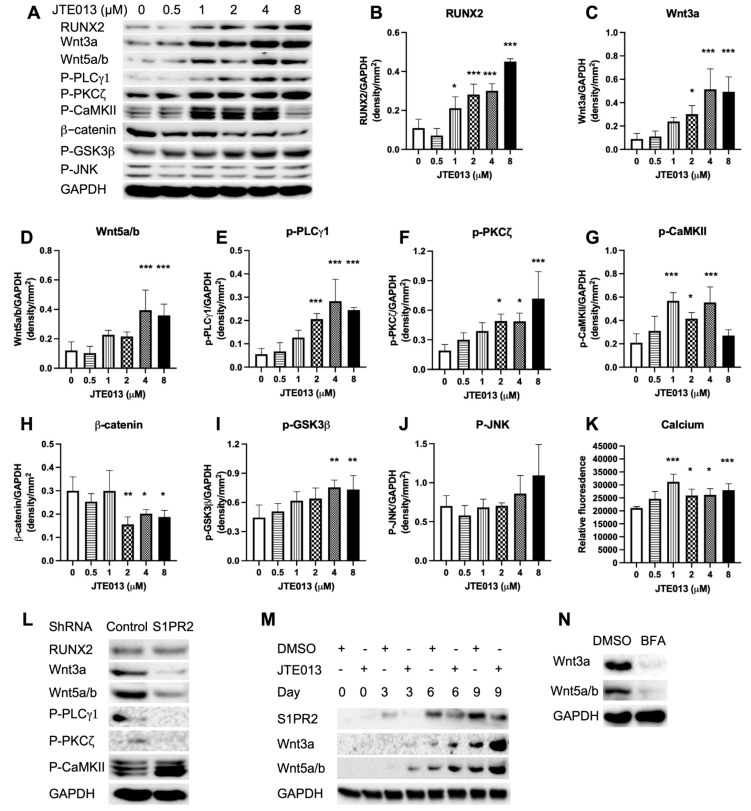
JTE013, but not a S1PR2 shRNA, increased RUNX2 and Wnt/Ca^2+^ signaling in murine BMSCs cultured in osteogenic media. (**A**) RUNX2, Wnt3a, Wnt5a/b, p-PLCγ1, p-PKCζ, p-CaMKII, β-catenin, p-GSK3β, p-JNK, and GAPDH protein expressions in murine BMSCs cultured in osteogenic media with either DMSO or JTE013 (0.5 to 8 μM) for 9 days. Proteins were harvested 30 min after changing osteogenic media with or without JTE013. Protein densitometry of (**B**) RUNX2, (**C**) Wnt3a, (**D**) Wnt5a/b, (**E**) p-PLCγ1, (**F**) p-PKCζ, (**G**) p-CaMKII, (**H**) β-catenin, (**I**) p-GSK3β, and (**J**) p-JNK was normalized by GAPDH protein expression in murine BMSCs (n = 3, * *p* < 0.05, ** *p* < 0.01, *** *p* < 0.001). (**K**) Ca^2+^ level in murine BMSCs treated with DMSO or JTE013 and cultured with osteogenic media for 9 days (n = 5, * *p* < 0.05, *** *p* < 0.001). (**L**) RUNX2, Wnt3a, Wnt5a/b, p-PLCγ1, p-PKCζ, p-CaMKII, and GAPDH protein expressions in BMSCs treated with a control shRNA or a S1PR2 shRNA and cultured in osteogenic media for 9 days. (**M**) S1PR2, Wnt3a, Wnt5a/b, and GAPDH protein expressions in murine BMSCs treated with DMSO or JTE013 (8 μM) from 30 min to 9 days. (**N**) Wnt3a, Wnt 5a/b, and GAPDH protein expressions in BMSCs treated with DMSO or brefeldin A (BFA, 0.75 μM) and cultured in osteogenic media for 6 days.

To determine how JTE013 and the S1PR2 shRNA influence signaling pathways that affect osteogenesis, we analyzed Wnt signaling pathways in murine BMSCs. Treatment with JTE013 (1 to 8 μM) significantly increased RUNX2, the master transcriptional factor for osteogenesis, in BMSCs compared with DMSO treatment (Figure 6A,B, * *p* < 0.05, ** *p* < 0.01, *** *p* < 0.001). We also noticed a dose-dependent increase in Wnt3a and Wnt5a/b protein expressions in JTE013-treated BMSCs compared with DMSO-treated cells (Figure 6A,C,D). Treatment with JTE013 (2 to 8 μM) significantly increased p-PLCγ1 and p-PKCζ in BMSCs compared with DMSO treatment (Figure 6A,E,F). JTE013 (1 to 4 μM) also significantly increased p-CaMKII compared with DMSO treatment (Figure 6A,G). These data support that JTE013 promoted osteogenesis via the Wnt/Ca^2+^ signaling pathway. In contrast, treatment with JTE013 (2 to 8 μM) significantly reduced the β-catenin protein level compared with DMSO treatment (Figure 6A,H). Treatment with JTE013 (4 to 8 μM) also enhanced the p-GSK3β protein level (Figure 6A,I), which plays a role in the degradation of β-catenin, compared with vehicle treatment. Additionally, there were no significant differences in p-JNK protein levels among treatment groups (Figure 6A,J), These data suggest that JTE013 promoted β-catenin degradation without affecting the Wnt/PCP pathway. Because treatment with a high dose of JTE013 (8 μM) failed to activate p-CaMKII (Figure 6A,G), we further quantified the calcium levels in BMSCs. As shown in Figure 6K, treatment with JTE013 (1–8 μM) significantly increased calcium release in BMSCs cultured with osteogenic media compared with DMSO treatment. Overall, these data demonstrate that JTE013 promoted osteogenesis via the Wnt/Ca^2+^ signaling pathway, not via the Wnt/β-catenin or Wnt/PCP pathways.

In murine BMSCs treated with the S1PR2 shRNA, the RUNX2 protein level was similar to the RUNX2 protein level in BMSCs treated with the control shRNA (Figure 6L). However, Wnt3a, Wnt5a/b, p-PLC, and p-PKC protein levels were attenuated compared with these protein levels in BMSCs treated with the control shRNA (Figure 6L). In contrast, p-CaMKII protein expression was elevated in murine BMSCs treated with the S1PR2 shRNA compared with BMSCs treated with the control shRNA (Figure 6L). These data suggest that the S1PR2 shRNA activated p-CaMKII independent of Wnt signaling pathway.

To determine when Wnts were elevated in murine BMSCs and when JTE013 promoted these Wnt protein expressions, we quantified Wnt3a and Wnt5a/b protein levels in murine BMSCs treated with either DMSO or JTE013 (8 μM) and cultured in osteogenic media from day 0 to day 9. On day 0 (30 min after treatment with either DMSO or JTE013), the S1PR2 protein level was very low. The S1PR2 protein level was gradually elevated from day 3 to day 9 in murine BMSCs cultured in osteogenic media (Figure 6M). JTE013 (8 μM) reduced S1PR2 protein levels in murine BMSCs cultured in osteogenic media from day 3 to day 9 (Figure 6M). On day 0, the Wnt3a and Wnt5a/b protein expressions were undetectable in murine BMSCs (Figure 6M). Wnt3a and Wnt5a/b proteins gradually increased in murine BMSCs cultured in osteogenic media from day 3 to day 9. JTE013 (8 μM) enhanced both Wnt3a and Wnt5a/b protein levels in BMSCs cultured in osteogenic media compared with BMSCs treated with DMSO from day 3 to day 9 (Figure 6M). To further determine whether Wnt3a and Wnt5a/b protein expressions were associated with vesicle trafficking, murine BMSCs were treated with brefeldin A (BFA, an inhibitor of protein trafficking from ER to Golgi) [46,47] or DMSO and cultured in osteogenic media for 6 days. Treatment with BFA suppressed both Wnt3a and Wnt5a/b protein expressions in BMSCs compared with DMSO treatment (Figure 6N). Overall, these data support that Wnt protein expressions were dependent on vesicle trafficking. JTE013 promoted vesicle trafficking and increased Wnt protein levels in murine BMSCs, whereas treatment with either BFA or the S1PR2 shRNA suppressed vesicle trafficking, and inhibited Wnt protein expressions in BMSCs.

### 2.7. Low Doses of JTE013 (1 to 2 μM), but Not the S1PR2 ShRNA, Promoted BMPs/Smad Signaling in Murine BMSCs

**Figure 7:**
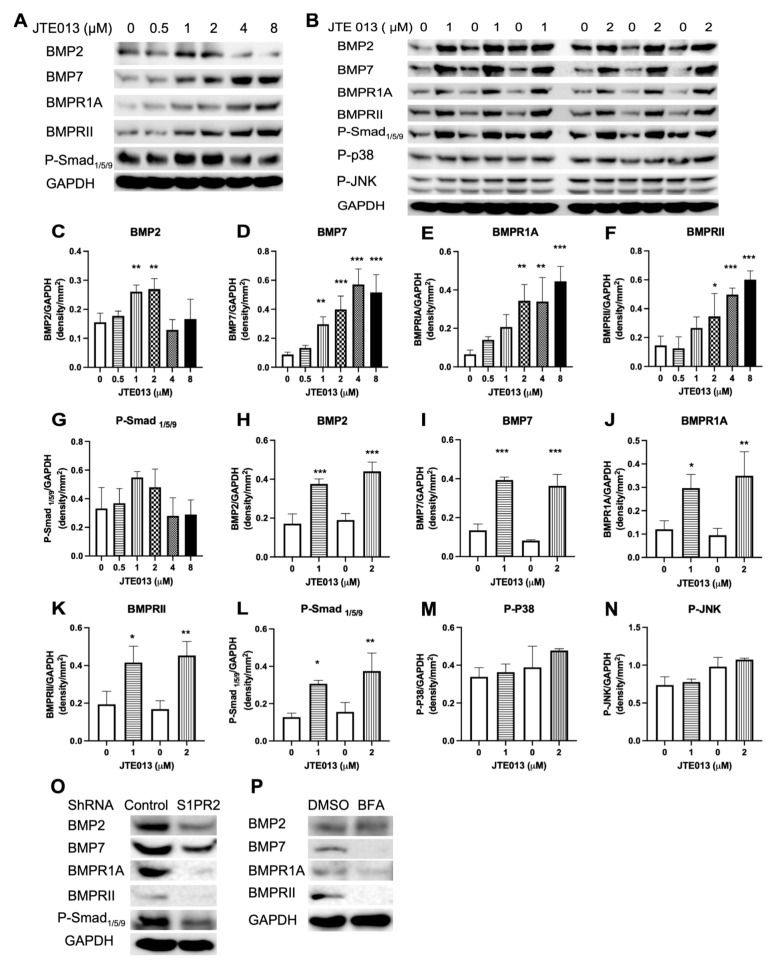
Low doses (1 to 2 μM) of JTE013, but not a S1PR2 shRNA, increased BMPs/Smad signaling in murine BMSCs cultured in osteogenic media. Murine BMSCs were cultured in osteogenic media with either DMSO or JTE013 for 9 days. Proteins were harvested 30 min after changing osteogenic media with or without JTE013. (**A**) BMP2, BMP7, BMPR1A, BMPRII, p-Smad_1/5/9_, and GAPDH protein levels in BMSCs treated with DMSO or JTE013 (0.5 to 8μM). (**B**) BMP2, BMP7, BMPR1A, BMPRII, p-Smad_1/5/9_, p-p38, p-JNK, and GAPDH protein levels in BMSCs treated with DMSO or JTE013 (1 or 2 μM). Protein densitometry of (**C**,**H**) BMP2, (**D**,**I**) BMP7, (**E**,**J**) BMPR1A, (**F**,**K**) BMPRII, (**G**,**L**) p-Smad_1/5/9_, (**M**) p-p38, and (**N**) p-JNK was normalized by GAPDH protein expression in BMSCs (n = 3, * *p* < 0.05, ** *p* < 0.01, *** *p* < 0.001). (**O**) BMP2, BMP7, BMPR1A, BMPRII, p-Smad_1/5/9_, and GAPDH protein levels in BMSCs treated with a control shRNA or a S1PR2 shRNA and cultured in osteogenic media for 9 days. (**P**) BMP2, BMP7, BMPR1A, BMPRII, and GAPDH protein levels in BMSCs treated with DMSO or brefeldin A (BFA, 0.75 μM) and cultured in osteogenic media for 6 days.

When we analyzed BMP signaling in BMSCs, we observed a significant increase in BMP2 (Figure 7A,C, ** *p* < 0.01) and a trend of increase in p-Smad_1/5/9_ (Figure 7A,G) in BMSCs treated with a low dose of JTE013 (1 or 2 μM) compared with DMSO treatment. Treatment with JTE013 also dose dependently increased BMP7, BMPR1A, and BMPRII protein levels in BMSCs compared with DMSO treatment (Figure 7A,D–F). To further confirm this finding, we treated BMSCs with either DMSO or JTE013 (1 or 2 μM). Treatment with low doses of JTE013 (1 to 2 μM) significantly increased BMP2, BMP7, BMPR1A, BMPRII, and p-Smad_1/5/9_ protein levels compared with DMSO treatment (Figure 7B,H–L). In contrast, there were no significant differences in MAPKs (including p-p38 and p-JNK) between JTE013-treated groups and DMSO-treated groups (Figure 7B,M–N). These data support that low doses of JTE013 (1 to 2 μM) specifically activated the BMPs/Smad signaling pathway, but not the BMPs/MAPK pathway.

In murine BMSCs treated with the S1PR2 shRNA, the protein levels of BMP2, BMP7, BMPR1A, BMPRII, and p-Smad_1/5/9_ were attenuated compared with those protein levels in BMSCs treated with the control shRNA (Figure 7O). These data suggest that the S1PR2 shRNA attenuated the BMP/Smad signaling pathway. In murine BMSCs treated with brefeldin A and cultured in osteogenic media for 6 days, brefeldin A suppressed BMP7, BMPR1A, and BMPRII, but not BMP2 protein expressions in BMSCs compared with murine BMSCs treated with DMSO (Figure 7P), which suggests that the protein synthesis and transport of BMP7, BMPR1A, and BMPRII might be involved in the ER–Golgi signaling pathway.

## 3. Discussion

In this in vitro study, we demonstrated that treatment with a S1PR2 specific antagonist (JTE013) or a S1PR2 shRNA increased ALP and ARS staining, and enhanced osteogenic gene (ALPL, RUNX2, OCN, OSX) expressions in murine BMSCs cultured in osteogenic media. Protein analysis revealed that treatment with JTE013 increased vesicle trafficking-associated proteins (F-actin, EEA1, clathrin, syntaxin 6) and enhanced Wnt/Ca^2+^ signaling-associated proteins (Wnt3a, Wnt5a/b, p-PLCγ1, p-CaMKII, and p-PKCζ). Additionally, low doses of JTE013 (1 to 2 μM) increased BMPs/Smad signaling-associated proteins (BMP2, BMP7, BMPR1A, BMPRII, and p-Smad_1/5/9_). In contrast, treatment with the S1PR2 shRNA inhibited vesicle trafficking-associated proteins (F-actin, EEA1, clathrin, syntaxin 6), reduced Wnt3a, Wnt5a/b, p-PLCγ1, and p-PKCζ protein levels, but increased the p-CaMKII protein level compared with the control shRNA treatment. Treatment with the S1PR2 shRNA also suppressed BMP2, BMP7, and p-Smad_1/5/9_ protein expressions compared with the control shRNA treatment. These data support that the S1PR2 shRNA acted on different cell signaling pathways in murine BMSCs. The activation of p-CaMKII was independent of Wnt protein expressions. We observed an increase of ARS (mineralization staining) in murine BMSCs treated with the S1PR2 shRNA (Figure 3D–E), which might be associated with the activation of p-CaMKII in BMSCs treated with the S1PR2 shRNA. Future studies are needed to determine how the S1PR2 shRNA regulates cell signaling pathways affecting osteogenesis.

It is well-known that vesicle trafficking plays an important role in bacterial endocytosis/phagocytosis [48] and synaptic vesicle release in neurological diseases [49]. However, little is known about the role of vesicle trafficking in osteogenesis. We are the first to emphasize that vesicle trafficking is an important signaling pathway in osteogenesis. In this in vitro experiment, vesicle trafficking was involved in the uptake of proteins and minerals from cell culture media, the synthesis and transport of the intracellular vesicle pool via endosomes and the Golgi network, and the exocytosis of proteins and matrix vesicles on the plasma membrane. Endosomes function to sort cargo proteins, lipids, and minerals [50]. Endosomal proteins and lipids can either be delivered to the cell surface via the recycling of the endosome pathway, or they can be transported to the Golgi network before reaching the lysosomes for degradation. The increased expressions of EEA1 and clathrin suggest that JTE013 promoted endocytosis in BMSCs cultured in osteogenic media. Additionally, clathrin is involved in exocytosis, as clathrin depletion blocked exocytosis [51]. Syntaxin 6, a SNARE (soluble N-ethylmaleimide sensitive factor activating protein receptor) protein, plays an essential role in vesicle fusion to transport cargos from endosomes to Golgi network as well as vesicle fusion with the plasma membrane to release intracellular vesicles. We observed a dose-dependent increase of ARS staining, a symbol of mineralization, in BMSCs treated with JTE013, which might be associated with the enhanced generation of matrix vesicles inside of BMSCs and increased exocytosis of matrix vesicles on the plasma membrane in JTE013-treated BMSCs.

Rac acts on multiple roles in vesicle trafficking. First, Rac can induce actin polymerization, which drives vesicle trafficking [43]. Second, Rac can directly activate PLC, leading to the generation of inositol triphosphate (IP3), which increases calcium release [52,53]. Calcium, in turn, plays an essential role in exocytosis. A majority of the secretory granules accumulated in the cytosol, creating a large, undocked vesicle pool, known as the reserve pool. Exocytosis undergoes a series of events: recruitment of vesicles to the plasma membrane, specific tethering at the plasma membrane, priming, and then triggering membrane fusion [54]. Calcium functions both in priming and vesicle fusion. The first priming step requires a moderate increase in Ca^2+^, as well as the presence of adenosine triphosphate magnesium, whereas a rapid substantial elevation of Ca^2+^ is required for vesicle fusion [54]. In this study, we observed an increased p-PLCγ1 protein expression and Ca^2+^ release, which might be associated with the increased Rac1-GTP in JTE013-treated BMSCs. Additionally, calcium influx triggers endocytosis because reducing or buffering calcium influx abolished endocytosis and reduced exocytosis [42]. The enhanced ARS staining in JTE013-treated BMSCs supports that more matrix vesicles were deposited on the plasma membrane of BMSCs treated with JTE013.

**Figure 8:**
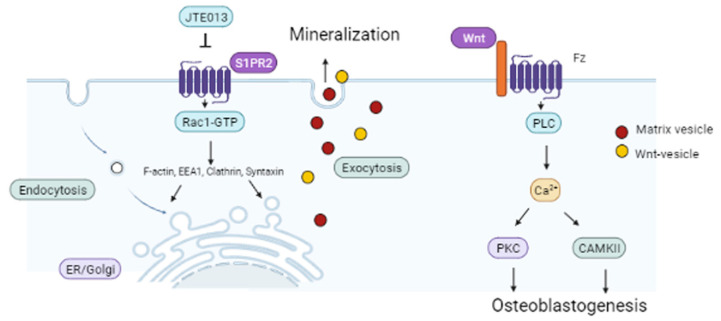
Schematic representation of the role of JTE013 in osteogenesis. Treatment with the S1PR2 antagonist, JTE013, increases Rac1-GTP, which promotes polymerization of actin to form F-actin and increases EEA1, clathrin, and syntaxin levels. These proteins, in turn, further stimulate both endocytosis and exocytosis. Exocytosis of Wnt vesicles excretes Wnt, which binds to the Fz receptor and activates PLC. Activation of PLC releases calcium, which serves as a second messenger to activate CaMKII and PKC. Activation of Wnt/Ca^2+^ signaling promotes osteoblastogenesis. Additionally, the exocytosis of matrix vesicles promotes mineralization.

Vesicle trafficking not only plays an important role in mineralization, but also is involved in protein synthesis and secretion, including with Wnts. Following synthesis, Wnt proteins are processed in the ER, where they undergo post-translational modification (an ER-localized enzyme catalyzes the transfer of palmitoleic acid onto Wnts) [55]. Wnt chaperone Wntless (Wls) binds to Wnts through its palmitoleic acid moiety to transport Wnts from the ER via the Golgi to the plasma membrane [55]. Following the delivery of Wnts to the plasma membrane, Wls undergoes endocytosis and is recycled back to the Wnt-producing cells, where it binds to newly synthesized Wnt proteins and traffics them to the plasma membrane [55]. Yamamoto et al. [56] demonstrated that the secretion of Wnt3a is attenuated by the depletion of clathrin, which suggests that endocytosis is involved in Wnt3a secretion. In this study, we observed a dose-dependent increase of Wnt3a and Wnt5a/b protein expressions in BMSCs treated with JTE013, which might be associated with increased endocytosis of Wls, as well as increased synthesis and export of Wnts in JTE013-treated BMSCs (Figure 8). In this study, BMSCs treated with the S1PR2 shRNA suppressed vesicle trafficking-associated protein levels (Figure 5H), and attenuated Wnt3a and Wnt5a/b protein levels (Figure 6L) compared with BMSCs treated with the control shRNA. Additionally, inhibition of vehicle trafficking by brefeldin A also suppressed Wnt3a and Wnt5a/b protein levels in murine BMSCs cultured in osteogenic media (Figure 6N). Our study results are congruent with those reported by Yamamoto et al. [56], supporting that Wnt protein expressions are dependent on vesicle trafficking.

In addition to Wls, BMP receptors also undergo endocytosis, subsequently influencing BMP signaling [57,58,59]. It has been demonstrated that the endocytosis of BMPRs is via clathrin-coated vesicles, cholesterol-rich lipid rafts, and caveolin-rich domains [57,58,59]. Increased vesicle trafficking allows for the recycling of BMPRs to the plasma membrane, which may support signal renewal [57]. Additionally, the endocytosis of BMPRs enables BMPRs to interact with other proteins that mediate the propagation of signaling to different intracellular locations [57,59]. Hartung et al. [58] used chemicals to deplete cholesterol or disrupt clathrin-mediated endocytosis, which did not affect the initial p-Smad expression. Instead, these chemicals reduced BMP2-induced ALP expression, which suggested that the continuation of Smad signaling requires BMPRs endocytosis [58]. In this study, we observed a dose-dependent increase of BMP7, BMPR1A, and BMPRII in JTE013-treated BMSCs (Figure 7A,D–F). BMSCs treated with the S1PR2 shRNA inhibited vesicle trafficking-associated proteins (Figure 5H) and reduced BMP7, BMPR1A, and BMPRII protein expressions in BMSCs compared with BMSCs treated with the control shRNA (Figure 7O). Additionally, inhibition of protein transport from the ER to Golgi by brefeldin A suppressed BMP, BMPR1A, and BMPRII protein expressions in BMSCs compared with the control (Figure 7P). These data suggest that the synthesis and transport of BMP7, BMR1A, and BMPRII might be involved in the ER–Golgi signaling pathway. We observed an increase of OCN mRNA levels on day 10 in BMSCs treated with JTE013 (1μM) (Figure 4G), which might be associated with enhanced BMPs/Smad signaling in these cells (Figure 7B,H–L).

Because S1PR2 couples with multiple G_i_, G_q_, and G_12/13_ proteins, with S1PR1 co-coupled to G_i_, S1PR3 co-coupled with G_i_, G_q_, and G_12/13_ proteins, and S1PR4 and S1PR5 co-coupled to G_i_ and G_12/13_ [1], other S1PRs (S1PR1, S1PR3, S1PR4, and S1PR5) might have overlapping and compensatory roles. In this study, we observed elevated expressions of both mRNA and protein levels of S1PR1, S1PR3, S1PR4, and S1PR5 in murine BMSCs treated with a high dose of JTE013 (4 or 8 μM). This might be caused by the stimulation of compensatory increase of these S1PRs by JTE013 or due to increased biosynthesis of these S1PRs via the ER–Golgi signaling pathway. A previous study [60] indicated that JTE013 was both an S1PR2 and S1PR4 antagonist because JTE013 (10 μM) was reported to inhibit S1P-induced p-ERK expression in MAD-MB-453 breast cancer cells, and a S1PR4 siRNA also reduced the S1P-induced p-ERK, while a S1PR2 siRNA failed to reduce the S1P-induced p-ERK expression in MDA-MB-453 breast cancer cells [60]. However, the study did not reveal S1PR2, S1PR4 mRNA, or protein levels in MDA-MB-453 breast cancer cells treated with JTE013. In our previous study [20], knockdown of S1PR2 by a S1PR2 shRNA in murine BMMs reduced p-ERK protein levels at 2 and 4 h that were induced by an oral bacterial pathogen (*Aggregatibacter actinomycetemcomitans*), which demonstrated that S1PR2 controls p-ERK expression. S1PR4 might have overlapping role in controlling p-ERK expression. In this study, JTE013 (4 to 8 μM) only reduced S1PR2 protein expression without attenuation of S1PR2 mRNA levels, which suggested that JTE013 possibly targets S1PR2 protein for degradation. Although both JTE013 (4 or 8 μM) and the S1PR2 shRNA reduced S1PR2 protein level in murine BMSCs, JTE013 promoted vesicle trafficking, while the S1PR2 shRNA inhibited vesicle trafficking. The increased vesicle trafficking in JTE013-treated cells might be caused by some off-target effects, including the effects caused by enhancing S1PR1, S1PR3, S1PR4, and S1PR5 mRNA and related protein levels in murine BMSCs (Figure 1E–O).

Because S1PR2 couples with multiple G_i_, G_q_, and G_12/13_ proteins, the inhibition of S1PR2 by JTE013 influences multiple signaling pathways. These signaling pathways could also cross talk to each other, thus affecting osteogenesis. Previous studies have demonstrated that the R-Smad linker region contains conserved MAPKs and GSK3 phosphorylation sites, which are crucial targets for regulating the stability of activated R-Smads [59,61]. Phosphorylation of the Smad1 linker by MAPKs and GSKs leads to Smad1 protein ubiquitination via Smurf1, a member of the E3 ubiquitin ligase family, resulting in Smad1 protein degradation [59,61]. GSK3 can also phosphorylate Smad4, leading to Smad4 ubiquitination and degradation [62]. Additionally, CaMKII phosphorylates Smad 2 and Smad4, and to a lesser extent, Smad3 [63]. PKC phosphorylates Smad 3 and Smad 2 [63]. In higher doses of JTE013, we observed an increase of p-GSK3β (Figure 6A,H), but lower activation of p-Smad_1/5/9_ (Figure 7A,G), which might be associated with increased phosphorylation of the link region of Smad_1/5/9_ by GSK3 or by other protein kinases leading to Smad_1/5/9_ ubiquitination and degradation. A previous study [64] also demonstrated that PKC phosphorylated N-terminal serine residues of β-catenin and promoted β-catenin degradation. In BMSCs treated with JTE013, we observed a dose-dependent increase of p-PKCζ and p-GSK3β (Figure 6A,F,I), which might have contributed to the degradation of β-catenin (Figure 6A,H) in this study. In BMSCs treated with JTE013 (8 μM), the p-CaMKII was not activated (Figure 6A,G). This might be associated with cross talk with other signaling pathways that affect CaMKII activation. Future studies are needed to determine which signaling pathways cross talk with Wnt/Ca^2+^, affecting CaMKII activation.

In this study, the results conflict with those from a previous study [14]. Previously, Higashi et al. [14] showed that treatment with JTE013 (10 μM) in either mouse osteoblast-like MC3T3-E1 cells or primary osteoblasts inhibited RUNX2 induced by S1P (2 μM). However, the constitution levels of S1P in most tissues are very low (10–30 nM) because S1P can be degraded either by S1P lyase or dephosphorylated by S1P phosphatase [2,4]. Therefore, using S1P (2 μM) to stimulate osteoblasts is an artificial effect. In this study, we only cultured BMSCs in osteogenic media without the addition of S1P. Second, when Higashi et al. [14] determined the RUNX2 protein level, they only cultured osteoblast cells in serum-free media for 24 h and then cultured cells in cell growth media with S1P (2 μM) for 24 h. In our study, we harvested protein from BMSCs cultured in osteogenic media with DMSO or JTE013 for 9 days. Osteogenesis from BMSCs is a long-term process. In the early stages (before day 3), osteogenic proteins (BMPs, RUNX2, Wnts, and β-catenin) were undetectable in BMSCs cultured in osteogenic media (data not shown). We observed a reduction of protein concentration in BMSCs treated with a high dose of JTE013 (4 or 8 μM) on day 9 (data not shown), which suggested that the high dose of JTE013 inhibited cell growth. However, because JTE013 enhanced Wnt protein expressions in BMSCs (Figure 6A,C,D), and because Wnts determine the fate of stem cell differentiation [26], we observed increased RUNX2 protein expression in JTE013-treated BMSCs (Figure 7A,B). Our ALP and ARS staining (Figure 2 and Figure 3) and our osteogenic genes’ (ALPL, RUNX2, OCN, OSX) expressions in BMSCs (Figure 4) also supported that JTE013 promoted the differentiation of osteoblasts. Higashi et al. [14] also demonstrated that C57BL/6N mice treated with a S1PR2 agonist, CYM5520, had enhanced bone volume in the tibia of mice. However, our results showed that CYM5520 had no effect on ALP staining and ARS staining in BMSCs cultured in osteogenic media (Figure 2 and Figure 3). Future studies are needed to determine how CYM5520 affects osteogenesis. This study results were consistent with Price et al.’s study [15], which showed that a low dose of JTE013 (1 μM) promoted the growth of murine BMSCs (Figure 1B). Price et al. [15] did not determine the effect of a high dose of JTE013 on osteogenesis. This study shows that only high doses of JTE013 (above 4 μM) significantly reduced S1PR2, while low doses of JTE013 (0.5 to 2 μM) failed to attenuate the S1PR2 protein level (Figure 1J–K).

Our understanding of the signaling pathways that regulate osteogenesis is evolving rapidly. Herein, we demonstrated that BMSCs treated with the S1PR2 antagonist, JTE013, enhanced osteogenesis, which is associated with promoting vesicle trafficking, Wnt/Ca^2+^, and BMPs/Smad signaling. We emphasize that vesicle trafficking plays an essential role in osteogenesis by increasing the intracellular biosynthesis of osteogenic proteins and mineral vesicles, and by exporting Wnts and matrix vesicles on the plasma membrane, thus promoting osteogenesis and bone matrix mineralization.

## 4. Materials and Methods

### 4.1. Cells and Reagents

Primary murine bone marrow stromal cells (BMSCs) were obtained from Dr. James Cray’s laboratory (the Ohio State University, Columbus, OH, USA). These BMSCs were isolated from femurs of several male adult CD-1 mice by Stem Cell Core, Augusta University (Augusta, Georgia, USA). The isolation of BMSCs was conducted using a protocol described previously [65]. Briefly, bone marrow cells were cultured in complete isolation media (RPMI-1640 culture media with 10% FBS and 100 U/mL penicillin and streptomycin) for 3 h. Then, the media containing non-adherent cells were removed. The adherence cells were washed with PBS and were cultured in the complete isolation media for 3–4 weeks with the media changed every 3–4 days. At 70–80% confluence, the cells were lifted by incubation with trypsin/EDTA. Then, the cells were selected for being positive to stem cell antigen 1 (Sca-1), but negative for anti-mouse CD11b, CD45R/B22O, and Pan DC monoclonal antibodies by using antibody-conjugated magnetic nanoparticle beads. These procedures removed monocytes, granulocytes, macrophages, dendritic cells, natural killer cells, B lymphocytes, T lymphocytes, and macrophage progenitors from BMSCs. BMSCs were grown in high glucose Dulbecco’s Modified Eagle Medium (DMEM), supplemented with 10% fetal bovine serum (FBS, USDA approved source, Fisher Scientific, Suwanee, GA, USA) and 100 U/mL penicillin and streptomycin. A passage number of 9 murine BMSCs were used in this study. JTE013 and CYM5520 were purchased from Cayman Chemical (Ann Arbor, MI, USA), dissolved in DMSO, and diluted using cell culture media. Dexamethasone, ascorbic acid, β-glycerophosphate, polybrene, alizarin red S, and a leukocyte alkaline phosphatase kit were purchased from Sigma Aldrich (St. Louis, MO, USA). DMSO, 2-amino-2-methyl-1-propanol (AMP), p-nitrophenol, puromycin, brefeldin A, p-nitrophenyl phosphate, acetic acid, ammonium hydroxide, DMEM, penicillin, and streptomycin were purchased from Fisher Scientific.

### 4.2. Osteogenic Induction

The osteogenic medium was high glucose DMEM, supplemented with 10% FBS, 10 mM β-glycerophosphate, 0.1 μM dexamethasone, 0.5 mM ascorbic acid, and 100 U/mL penicillin and streptomycin. First, 5 × 10^4^ BMSCs were placed in each well of 24-well plates (for ALP and ARS assay, protein quantification, and RNA extraction) or 4 × 10^5^ BMSCs were placed in 60 mm dishes (for Western blot assay) and cultured in high glucose DMEM, supplemented with 10% FBS and 100 U/mL penicillin and streptomycin for 24 h. The next day, the cell culture media were replaced with osteogenic media. The osteogenic medium was changed every 2 days. Because osteoblastogenesis is a long-term process, murine BMSCs were cultured in osteogenic media with the presence of either JTE013, DMSO, a S1PR2 shRNA, or a control shRNA from 30 min to 21 days.

### 4.3. Cell Proliferation Assay

For the cell proliferation assay, 1 × 10^4^ BMSCs were placed in each well of 96-well plates in high glucose DMEM media supplemented with 10% FBS and 100 U/mL penicillin and streptomycin. The second day, the cell culture media were changed with osteogenic medium containing either DMSO or JTE013 (0.5 to 8 μM) for 24 to 72 h. Cell growth was quantified by a CellTiter 96^®^ AQueous One Solution Cell Proliferation Assay (Promega, Madison, WI, USA).

### 4.4. ALP Staining and Quantification

BMSCs were fixed with 10% buffered formalin (Fisher Scientific) for 1 min at room temperature (RT). After washing with deionized water once, the cells were stained in alkaline-dye solution (prepared from a leukocyte alkaline phosphatase kit according to Sigma Aldrich’s instructions) at RT for 30 min. After removing the dye solution, the BMSCs were washed with deionized water twice and photographed. The ALP quantification was performed according to a previous study [66]. Briefly, BMSCs were lysed in 200 μL lysis buffer (containing 25 mM Tris-HCL with 0.5% Triton X-100) at 4 °C for 2 h. After scraping and collecting the lysate in 1.5 mL tubes, the cells were vortexed for 1 min and centrifuged at 2500× *g* for 15 min at 4 °C. Then, 50 μL of supernatant derived from each sample was transferred to a new well of the 96-well plate, mixed with 50 μL of 50 mM p-nitrophenyl phosphate in AMP buffer, and incubated at 37 °C in an incubator for 30 min. A 10 times serial dilution of p-nitrophenol (stock 5000 μM) diluted in AMP buffer was used as standard curve. The amount of released p-nitrophenol was measured at an absorbance of 405 by a VersaMax microplate reader (Molecular Devices, San Jose, CA, USA). The OD405 value was calibrated by protein concentration in cell protein lysates.

### 4.5. ARS Staining and Quantification

BMSCs were fixed with 10% buffered formalin for 20 min at RT. After washing with deionized water once, the cells were stained in 40 mM ARS solution (pH 4.2) for 20 min. After aspirating the staining solution, the cells were washed with deionized water three times and photographed. The ARS quantification was performed as reported previously [67]. Briefly, BMSCs were incubated with 300 μL of 10% acetic acid at RT for 30 min with shaking. After scraping and collecting the cell lysate in a 1.5 mL tube, the cells were vortexed for 30 s. The slurry was overlaid with 250 μL mineral oil, heated at 85 °C for 10 min, and transferred to ice for 5 min. Then the slurry was centrifuged at 20,000× *g* for 15 min. Next, 250 μL of supernatant was transferred to a new 1.5 mL tube and mixed with 100 μL of 10% ammonium hydroxide to neutralize the acid. After adjusting the pH value of mixture to 4.2, aliquots of 100 μL of the mixture were loaded in triplicate in a 96-well plate. A 10 times serial dilution of ARS solution was used as standard curve. The absorbance 405 was detected by the VersaMax microplate reader and calibrated by the protein concentration in cell protein lysates.

### 4.6. A S1PR2 shRNA or a Control shRNA Lentiviral Vector Treatment

The S1PR2 shRNA and control shRNA lentiviral vectors were constructed and purified as previously described [20]. 4 × 10^5^ murine BMSCs were infected with either the S1PR2 shRNA lentiviral vector or the control shRNA lentiviral vector (multiplicity of infection 25) with the presence of polybrene (10 μg/mL) in high glucose DMEM media with 10% FBS and 100 U/mL penicillin and streptomycin. Then, 24 h after the lentiviral infection, the media were changed with high glucose DMEM media with 10% FBS, 100 U/mL penicillin and streptomycin, and puromycin (2 μg/mL) for 1 day. Then, the media were changed with osteogenic media with puromycin (2 μg/mL) for another 2 days and later changed with osteogenic media with puromycin (1 μg/mL) for two weeks. The osteogenic media were changed every two days.

### 4.7. RNA Extraction and Real-Time Polymerase Chain Reaction (PCR)

Total RNA was isolated from cells using TRIZOL (Life Technologies, Carlsbad, CA, USA) according to the manufacturer’s instructions. Complementary DNA was synthesized by a TaqMan reverse transcription kit (Life Technologies) using random hexamers and total RNA (1 μg). Each reverse transcription cDNA sample (20 μL) was diluted 5 times by ultra-pure distilled water and 5 μL of diluted cDNA was used for each real-time PCR reaction. Real-time PCR was performed using a StepOnePlus Real-Time PCR System (Life Technologies) and TaqMan gene expression master mix (Life Technologies). PCR conditions for this study were as follows: 50 °C for 2 min, 95 °C for 10 min, 40 cycles of 95 °C for 15 s, and 60 °C for 1 min. The following amplicon primers were obtained from Life Technologies: S1PR1 (Mm02619656_s1), S1PR2 (ARFVPA4), S1PR3 (Mm02620181_s1), S1PR4 (Mm00468695_s1), S1PR5 (mM02620565_s1), ALPL (Mm00475834_m1), RUNX2 (Mm00501584_m1), OCN (Mm03413826_mH), OSX (Mm04209856_m1), and GAPDH (Mm99999915_g1). The amplicon concentration was determined using threshold cycle values compared with standard curves for each primer. Sample mRNA levels were normalized to an endogenous control GAPDH expression and were expressed as fold changes as compared with control groups.

### 4.8. Protein Isolation and Western Blot Analysis

Proteins were extracted by RIPA cell lysis buffer (Cell signaling Technology, Danvers, MA, USA). The protein concentration was determined by a DC^TM^ protein assay kit (Bio-Rad Laboratories, Hercules, CA, USA). Rac1-GTP was analyzed by a Rac1 pull down activation assay biochem kit (Cytoskeleton, Inc., Denver, CO, USA). For the analysis, 25 μg of protein was loaded on 10% Tris-HCl gels and electro-transferred to nitrocellulose membranes. Membranes were blocked with milk for 1 h at RT and incubated with the primary antibody overnight at 4 °C. The antibodies of the clathrin heavy chain, early endosome antigen 1 (EEA1), syntaxin 6, Runx2, Wnt3a, Wnt5a/b, p-PLCγ1, p-PKCζ, p-CaMKII, β-catenin, p-GSK3β, p-JNK, p-p38, p-Smad 1/5/9, and glyceraldehyde-3-phosphate dehydrogenase (GAPDH) were purchased from Cell Signaling Technology (Danvers, MA, USA). Antibodies of F-actin, BMP2, and BMPR1A were obtained from Abcam (Cambridge, MA, USA). Antibodies of BMP7 and BMPRII were purchased from Santa Cruz Biotechnology Inc. (Dallas, TX, USA). The S1PR1 antibody (PA1-1040), S1PR3 antibody (PA5-23225), S1PR4 antibody (PA5-102054), and S1PR5 antibody (PA5-98877) were all purchased from Thermo Fisher Scientific (Waltham, MA, USA). The S1PR2 antibody (SAB4503614) was obtained from Sigma Aldrich. All primary antibodies were incubated at 1:500 to 1:1000 dilution overnight at 4 °C. After washing, the nitrocellulose membranes were incubated at RT for 1.5 h with horseradish peroxidase-conjugated secondary antibodies (1:1000, Cell Signaling Technology) and developed using SuperSignal West Pico Chemiluminescent Substrate (Life Technologies, Carlsbad, CA, USA). Digital images were recorded by a G-BOX chemiluminescence imaging system (Syngene, Frederick, MD, USA). Protein densitometry was analyzed by the GeneTools software (Syngene) and normalized by GAPDH expression.

### 4.9. Immunofluorescence Staining

2.5 × 10^4^ BMSCs were placed on a coverslip in 35 mm dishes in high glucose DMEM media with 10% FBS. After 4 h, BMSCs were starved by being treated with high glucose DMEM media with 1% FBS overnight. The second day, the cell culture media were changed to osteogenic media with DMSO or JTE013 (8 μM) for 30 min. BMSCs were fixed by 4% paraformaldehyde for 10 min at RT. After three washes with PBS, the cells were permeabilized by treatment with 0.1% Triton X-100 in PBS for 10 min at RT. After another three washes with PBS, the BMSCs were blocked with 5% goat serum for 1 h at RT. Following another three washes with PBS, the BMSCs were incubated with anti-clathrin heavy chain (1:100, Cell Signaling Technology) with 1% bovine serum albumin (BSA) overnight at 4 °C. Cells were washed again with PBS, and incubated with an Alexa fluor 488-labeled goat anti-rabbit secondary antibody (1:400, Fisher Scientific, Suwanee, GA, USA) with 1% BSA for 1 h at RT. After washing with PBS, BMSCs were incubated with rhodamine phalloidin (1:140, Cytoskeleton, Inc., Denver, CO, USA) with 1% BSA for 1 h at RT. Cells were washed again with PBS and mounted on a slide with Prolong Gold anti-fade reagent with DAPI (Fisher Scientific) overnight at RT. Digital images were recorded by a Zeiss Axio Imager A1 fluorescent microscope.

### 4.10. Calcium Quantification

The calcium content in BMSCs was measured by a Fluo-4 NW Calcium Assay kit (Thermo Fisher Scientific, Waltham, MA, USA). The fluorescence signal was recorded by a FLUOstar OPTIMA microplate reader (BMG LABTECH Inc., Cary, NC, USA).

### 4.11. Statistical Analysis

Data were analyzed by one-way ANOVA with Dunnett’s multiple comparisons test. All statistical tests were performed using GraphPad Prism software (GraphPad Software Inc., La Jolla CA, USA). Values are expressed as means ± standard error of the means (SEM) of three independent experiments. A *p* value of 0.05 or less was considered significant.

## Data Availability

The data presented in this study are available upon request from the corresponding author.
